# Measurement of the total angiotensinogen and its reduced and oxidised forms in human plasma using targeted LC-MS/MS

**DOI:** 10.1007/s00216-018-1455-2

**Published:** 2018-11-21

**Authors:** Lina A. Dahabiyeh, David Tooth, Robin W. Carrell, Randy J. Read, Yahui Yan, Fiona Broughton Pipkin, David A. Barrett

**Affiliations:** 1 Department of Pharmaceutical Sciences, School of Pharmacy, The University of Jordan, Amman, 11942 Jordan; 2 Centre for Analytical Bioscience, Advanced Materials and Healthcare Technologies Division, School of Pharmacy, University of Nottingham, University Park, Nottingham, NG7 2RD UK; 3 School of Life Sciences, Centre for Biomolecular Sciences, University of Nottingham, Nottingham, NG7 2RD UK; 4 Department of Haematology, Cambridge Institute for Medical Research, University of Cambridge, Hills Road, Cambridge, CB2 0XY UK; 5 Department of Clinical Biochemistry, Cambridge Institute for Medical Research, University of Cambridge, Hills Road, Cambridge, CB2 0XY UK; 6 Department of Obstetrics and Gynaecology, City Hospital, University of Nottingham, Nottingham, NG5 1PB UK

**Keywords:** Angiotensinogen, Redox switch, Pre-eclampsia, LC-MS/MS, Cys18, Marker peptide

## Abstract

Angiotensinogen (AGT) is a critical protein in the renin-angiotensin-aldosterone system and may have an important role in the pathogenesis of pre-eclampsia. The disulphide linkage between cysteines 18 and 138 has a key role in the redox switch of AGT which modulates the release of angiotensin I with consequential effects on blood pressure. In this paper, we report a quantitative targeted LC-MS/MS method for the reliable measurement of the total AGT and its reduced and oxidised forms in human plasma. AGT was selectively enriched from human plasma using two-dimensional chromatography employing concanavalin A lectin affinity and reversed phase steps and then deglycosylated using PNGase F. A differential alkylation approach was coupled with targeted LC-MS/MS method to identify the two AGT forms in the plasma chymotryptic digest. An additional AGT proteolytic marker peptide was identified and used to measure total AGT levels. The developed MS workflow enabled the reproducible detection of total AGT and its two distinct forms in human plasma with analytical precision of ≤ 15%. The LC-MS/MS assay for total AGT in plasma showed a linear response (*R*^2^ = 0.992) with a limit of quantification in the low nanomolar range. The method gave suitable validation characteristics for biomedical application to the quantification of the oxidation level and the total level of AGT in plasma samples collected from normal and pre-eclamptic patients.

**Electronic supplementary material:**

The online version of this article (10.1007/s00216-018-1455-2) contains supplementary material, which is available to authorized users.

## Introduction

**Fig. 1:**
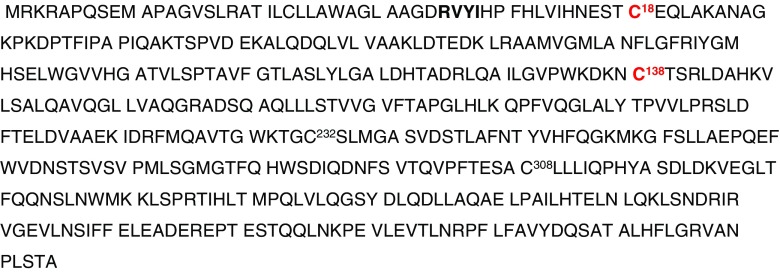
Amino acid sequence of AGT protein obtained from Swiss-Prot database. Cys18 and Cys138 are highlighted in bold red font. Sequence starts from RVYI…. presented in bold black letters; the first 33 amino acids are for the signal peptide

Cysteine (Cys) thiol plays a central role in protein structure and redox signalling, and this importance is highlighted by its involvement in many pathological conditions involving oxidative stress [1], including neurodegenerative diseases [2] and pre-eclampsia [3]. Angiotensinogen (AGT), Fig. 1, is a plasma glycoprotein and an essential component of the renin-angiotensin-aldosterone system (RAAS). The tail of AGT is cleaved by the action of the enzyme renin, in response to lowered blood pressure or increased sodium load at the macula densa, to yield the decapeptide angiotensin I, which is further cleaved by angiotensin converting enzyme (ACE) to generate the physiologically active octapeptide angiotensin II resulting in an increase in blood pressure [4]. The crystal structure of AGT revealed a key role for the disulphide linkage between Cys18 and 138 in the redox switch of AGT that modulates angiotensin release and, hence, potentially, blood pressure. Normally, the ratio between the free thiol unbridged form (reduced form) to the sulphydryl-bridged form (oxidised form) of AGT is maintained in the circulation at 40:60 respectively [3]. The oxidised form of AGT, compared to the free thiol form, interacts with renin with fourfold higher binding affinity resulting in an increased generation of angiotensin I and subsequently angiotensin II [3].

The crucial role of AGT in the RAAS has linked it with the pathogenesis of pre-eclampsia, a multisystem pregnancy disorder characterised by hypertension and proteinuria developing after the 20th week of gestation [5] and one of the leading causes of maternal and fetal mortality and morbidity worldwide [6, 7]. Only two studies have revealed a significant decrease in the level of the reduced form of AGT (reflecting a higher level of the oxidised form) in the plasma of pre-eclamptic women compared to controls [3, 8], which could be linked functionally to the increase in the blood pressure in pre-eclampsia. Previously, due to the lack of specific antibodies for the two distinct forms of AGT, prior quantification of the oxidation level of AGT relied on the alkylation of the reduced form of the protein and the use of a general antibody for AGT (not specific for each form of AGT) [3, 8], which can lead to inaccurate quantification. Although antibody-based assays are highly sensitive and are available in convenient high-throughput platforms, they suffer from poor selectivity to discriminate between different protein isoforms [9]. Additionally, the process of developing a highly selective antibody for a specific protein isoform is very challenging, expensive and time consuming [10, 11]. Therefore, there is a need for an entirely new strategy that enables the detection and the quantification of the two distinct forms of AGT, in the plasma, with high sensitivity and selectivity.

**Fig. 2:**
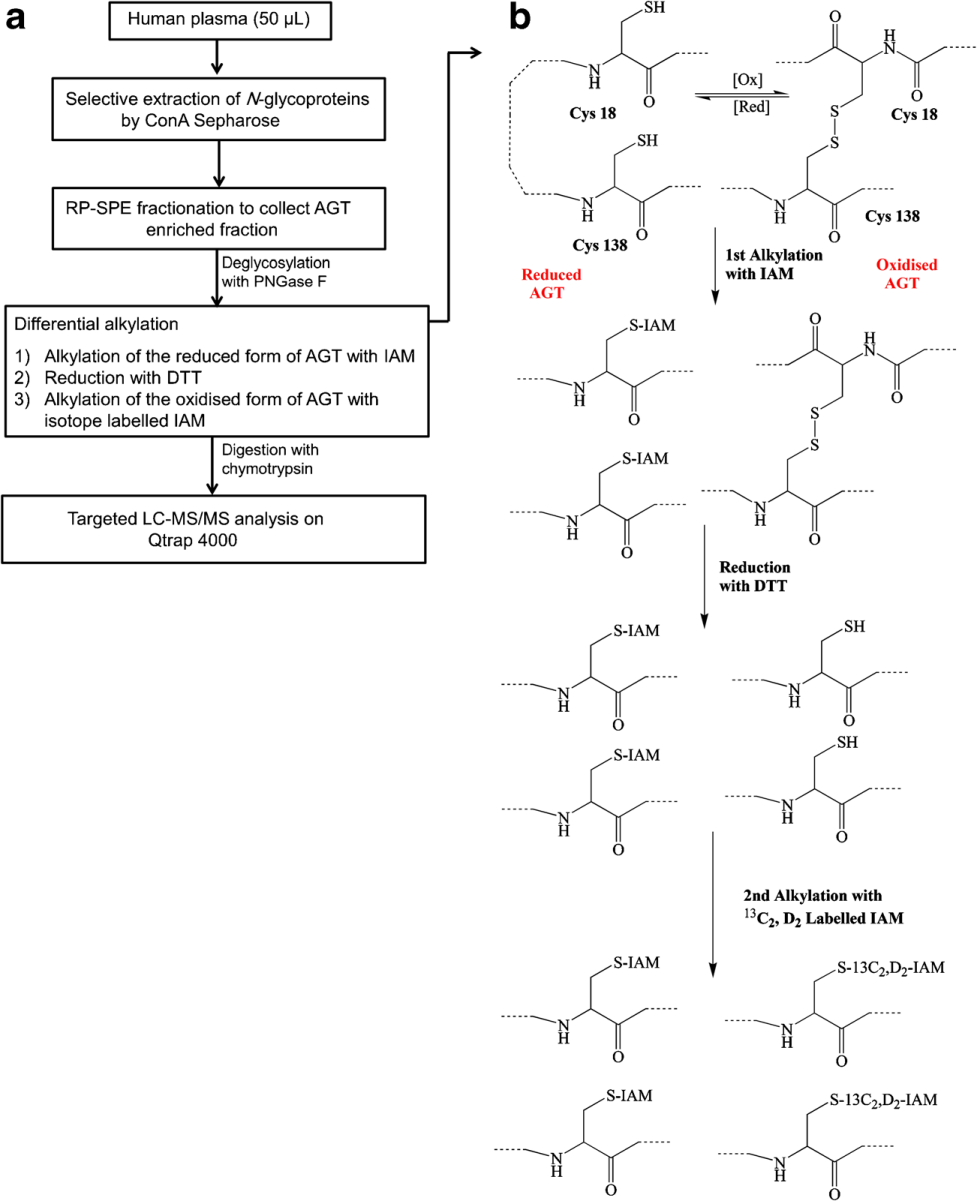
**a** Schematic representation of the workflow followed in this paper. Selective enrichment of AGT from human plasma was achieved using ConA Sepharose followed by fractionation with RP-SPE. The dried, collected RP-SPE fraction, containing AGT, was first deglycosylated with PNGase F followed by differential alkylation and finally digested using chymotrypsin. A targeted LC-MS/MS method was used to quantify signature peptides indicative of total AGT plus its oxidised and reduced forms in the plasma. **b** A diagram for the differential alkylation of the reduced and oxidised AGT using iodoacetamide (IAM) and isotope-labelled IAM

Mass spectrometry (MS) is a well-recognised analytical technique for protein identification, quantification and post-translational modification characterisation including disulphide linkage [12–14]. Targeted protein quantification by MS offers several advantages over immunoassay methods. It is highly multiplex and has shorter assay development timelines, lower assay cost, improved reproducibility and precision and, of most importance, higher specificity, which allows different structurally related protein isoforms to be selectively measured [15]. The advances in MS-based protein analysis techniques, including a range of variable redox analysis methodologies such as redox differential gel electrophoresis [16], isotope-coded affinity tags (ICAT) and differential alkylation [17, 18], and the availability of several Cys-selective chemical probes have considerably advanced redox protein analysis, and provided researchers with the tools and workflows necessary to assess thiol redox changes in complex biological systems [12–14]. Differential alkylation is the most common approach followed for the identification and quantification of reversible cysteine oxidations in proteins [17, 18]. In this approach, an initial irreversible alkylation of all free thiols with the first alkylating agent is carried out to avoid any unwanted thiol–disulphide exchange reactions that could affect the assessment of the oxidation status. A subsequent reduction of the disulphide bond and modification of the free thiols with a second alkylating agent uniquely identifies the two protein states [18] (Fig. 2b). Thiol-specific alkylation reagents (e.g. iodoacetamide (IAM) and N-ethylmaleimide (NEM)) can be conjugated to a wide variety of fluorophores and epitope tags for enrichment and detection of the alkylated Cys. Stable-isotope-labelled alkylating agents (e.g. d_5_-NEM, d_4_-IAM) are also available which enhances the use of the differential alkylation approach for redox protein detection and quantification [19].

The crucial role of the ratio of the two AGT forms in the pathogenesis of pre-eclampsia and the lack of a reliable and selective quantitative method that enables the identification and the quantification of the oxidised and the reduced AGT forms prompted us to develop and optimise, for the first time, an MS-based method for the reliable detection of chymotryptic peptides specific to the two AGT forms. We report here a sample preparation workflow specific for the selective enrichment and digestion of AGT from human plasma. A differential alkylation approach was coupled with targeted LC-MS/MS to detect the oxidised and the reduced forms of AGT in human plasma samples which were not detected by previous antibody-based strategies.

## Materials and methods

### Selective enrichment of AGT by two-dimensional chromatography using ConA lectin affinity and reversed-phase modes

Glycoproteins were first extracted using lectin affinity chromatography. Concanavalin A-Sepharose 4B (ConA, Sigma-Aldrich, UK) resin slurry (100 μL) was placed into micro-tube filter devices (Spin-X®, Corning Inc.) to provide a 1:1 ratio of resin bed to sample volume, and conditioned with 3 × 0.3 mL washing buffer (50 mM TRIS pH 7.4, 0.15 M NaCl, 1 mM CaCl_2_, MgCl_2_, MnCl_2_). The EDTA plasma samples (50 μL) were diluted in 250 μL washing buffer, loaded onto the conditioned resin and incubated at room temperature for 20 min with agitation. The resin was washed three times with the washing buffer to remove unbound proteins, and retained proteins were eluted with 1.2 mL elution buffer (0.40 M methyl α-D-mannopyranoside in washing buffer) for subsequent reversed-phase solid-phase extraction (RP-SPE) fractionation. The flow through the filter device was driven by centrifugation at 1000×*g* for 30 s in all steps.

Reversed-phase chromatography using RP-SPE was applied to extract AGT from glycoprotein plasma extract. Polymeric large-pore RP-SPE cartridges (IST ISOLUTE, PDVB, C18 1000 Å, 25 mg) were wetted with 1 mL of 0.1% (*v*/*v*) TFA in 90% acetonitrile prior to conditioning with 1 mL of 0.1% TFA in water. The enriched glycoprotein samples were loaded onto the preconditioned SPE cartridge and washed with 1 mL 0.1% TFA. The retained proteins were sequentially eluted with 1 mL of 30, 35, 40, 43, 45, 48, 50, and 90% (*v*/*v*) acetonitrile in 0.1% TFA in water, and the collected fractions were dried using vacuum centrifugation [20]. Human blood plasma was obtained with informed consent following approval from the School of Pharmacy Research Ethics Committee, University of Nottingham, UK.

### Monitoring AGT fractionation by Western blotting

The RP-SPE dried protein fractions were solubilised in SDS-PAGE sample buffer (NuPAGE Fisher Scientific, Loughborough, UK), and 20% of extracts were loaded onto 4–12% Bis TRIS midi-format gels and electrophoresed according to vendor guidelines. Proteins were electro-blotted in TRIS-glycine buffer (20% methanol, 50 mM pH 7.6) to polyvinylidene difluoride (PVDF) membrane. Immuno-detection of human plasma AGT in RP-SPE fractions by Western blotting used mouse anti-human serpin A8/angiotensinogen antibody (MAB3156, R&D Systems, UK) with horseradish-peroxidase-labelled anti-mouse IgG, and visualised with tetramethylbenzidine colourimetric reagent (Sigma-Aldrich, UK).

### Deglycosylation and chymotryptic digestion of enriched fractions of plasma AGT

The dried collected RP-SPE fractions containing AGT were reconstituted in 50 μL 2 M urea in TRIS buffer (pH 7.4), and proteins were deglycosylated using 1 μL PNGase F (P0705, 500,000 units/mL, glycerol free, New England Biolabs, UK) for each 40 μg proteins. Deglycosylation was carried out for 1.5 h at 37 °C followed by alkylation of reduced AGT with 25 μL 200 mM IAM. The oxidised form of AGT was reduced first with 25 μL 50 mM DTT, and the free thiol was then blocked with 25 μL ^13^C_2_,D_2_-IAM (98 atom% D, 99 atom% ^13^C, from Sigma-Aldrich, UK) to make the two AGT forms mass distinguishable. All reactions were carried out at 37 °C for 30 min in the dark using filter-assisted preparation (FASP) (10 kDa Amicon Ultra 0.5, Sigma-Aldrich, UK). After each reaction, the sample was washed twice with 400 μL of 50 mM ammonium hydrogen carbonate at 14,000×*g* for 7 min. Prior to digestion, the resulting 40 μL protein solution was diluted to 100 μL with the same buffer. Protein digestion was carried out using sequencing grade chymotrypsin (V106A, Promega, UK) at 37 °C using an enzyme: protein ratio of 1:50 (*w*/*w*). After 4 h, a second aliquot of chymotrypsin (1:100 enzyme to rotein *w*/*w*) was added and digestion proceeded at 37 °C overnight. The digestion reaction was quenched with 1% formic acid, and NEM alkylated Cys18 peptide was added to 0.2 μM net concentration as an internal standard. The samples were then centrifuged for 5 min at 11,000×*g*, and the upper 90 μL digest extract was submitted for LC-MS/MS analysis.

### Detection of the oxidised and reduced forms of AGT in the processed plasma samples using targeted LC-MS/MS

Chymotrypsin produced signature peptides were analysed using a 4000 QTRAP hybrid triple quadrupole/linear ion trap mass spectrometer (Applied Biosystems, Foster City, CA, USA) operating in positive ion mode. Peptides (10 μL of digest extract) were first separated by a Shimadzu series 10AD VP LC system (Shimadzu, Columbia, MD) using a C18, 300 Å, 100 × 1 mm, 3 μm column (ACE, Reading, UK) and a mobile phase of water and acetonitrile both with 0.1% formic acid. A gradient from 10% to 45% (*v/v*) acetonitrile was developed over 10 min at 100 μL/min flow rate.

Synthetic peptides corresponding to unmodified and IAM alkylated Cys18, Cys138 and AGT marker (Pierce Protein Biological Products, Thermo Fisher Scientific, UK) were used to optimise MS parameters and multiple reaction monitoring (MRM) transitions for each peptide. Standard synthetic peptides (2 μM) were infused at 10 μL/min flow rate to optimise the entrance and the declustering potentials for the three precursor ions and to choose the best three MRM transitions per peptide. The collision energy and cell exit potential for each MRM channel was optimised to maximise signal, and the most reproducible product ions with high signal intensity were chosen. Double-charged precursor ions were selected for the two modified Cys peptides but a single-charge precursor ion for the marker peptide. Source gases and temperature were optimised with a T piece in which an infusion of peptide standards was mixed with 0.1% formic acid acetonitrile (30%) eluting from the column at 100 μL/min flow rate. The optimised values for curtain gas, collision gas, GS1 and GS2 were 10, 12, 30 and 45 (arbitrary unit) respectively. The heated capillary temperature was maintained at 450 °C and the ESI voltage was kept at 4200 V.

MRM transitions were monitored and acquired at unit resolution in both Q1 and Q3 for the following AGT signature peptides: (1) AGT marker peptide (SVTQVPF), which was used to infer the plasma level of total AGT; (2) unlabelled-IAM alkylated Cys18 and Cys138 peptides, which correspond to the reduced form of plasma AGT and (3) ^13^C_2_,D_2_-IAM alkylated Cys18 and Cys138 peptides, which correspond to the oxidised form of plasma AGT. The final optimised transitions are presented in Table 1. All data were processed by Analyst software 1.4.2.

**Table 1:** The optimised precursor/product ion transitions for iodoacetamide and isotope-labelled iodoacetamide alkylated AGT Cys peptides and the total AGT marker peptide

Peptide sequence	Peptide mass	*m*/*z* (charge)	MS1/MS2	Product ion	DP	CE
HLVIHDEST**C**^**18**^EQL^a^ (IAM alkylated, reduced form)	1579.75	790.87 (+ 2)	790.9/725.3	b_12_^+2^	80	36
			790.9/844.4	b_7_	80	40
			790.9/1321.6	b_11_	80	38
			790.9/661.3	b_11_^+2^	80	40
HLVIHDEST**C**^**18**^EQL^a^ (isotope-labelled IAM, oxidised form)	1583.75	792.87 (+ 2)	792.9/727.3	b_12_^+2^	80	36
			792.9/844.4	b_7_	80	40
			792.9/1325.6	b_11_	80	38
			792.9/663.3	b_11_^+2^	80	40
KDKD**C**^**138**^TSRL^a^ (IAM alkylated, reduced form)	1121.57	561.78 (+ 2)	561.8/751.3	y_6_	65	38
			561.8/994.4	y_8_	65	32
			561.8/879.4	y_7_	65	34
			561.8/636.5	y_5_	65	38
KDKD**C**^**138**^TSRL^a^ (isotope-labelled IAM, oxidised form)	1125.57	563.78 (+ 2)	563.8/755.3	y_6_	65	38
			563.8/998.4	y_8_	65	32
			563.8/883.4	y_7_	65	34
			563.8/640.5	y_5_	65	38
SVTQVPF (AGT marker peptide)	776.41	777.41 (+ 1)	777.4/515.3	b_5_	75	32
			777.4/497.7	b_5_-H_2_O	75	30
			777.4/480.3	b_5_-2H_2_O	75	38

*CE* collision energy, *DP* declustering potential, *IAM* iodoacetamide, *MS1/MS2* precursor/product ions

^a^Peptide sequence after PNGase F processing contains Asp (D) instead of the native Asn (N) due to deglycosylation reaction

The identities of the targeted peptides were confirmed under similar chromatographic conditions on an ion trap mass spectrometer coupled to UPLC (LTQ Velos, Thermo Fisher, San Jose, CA, USA). MS/MS spectra of the peptides of interest were acquired in positive ion data-dependent mode with parent mass width ± 0.5 and retention time window 1 min. ESI source parameters were maintained as follows: ion spray voltage 3 kV, capillary and source heater temperatures 275 °C and 300 °C respectively and flow rate for the sheath, auxiliary and sweep gases 30, 20 and 10 arbitrary units respectively. Data were processed with Xcalibur 2.2 software (Thermo Fisher Scientific, San Jose, CA, USA).

### Method validation

Quantitative assessment of the developed LC-MS/MS method was carried out by measuring the precision (expressed as coefficient of variation, CV%) of six replicate human plasma samples (for the five monitored peptides). Precision values of ≤ 15% were considered acceptable, in accordance with the US-FDA bioanalytical method validation, guidance for industry, May 2018 [21].

The overall AGT recoveries were calculated by comparing the peak areas of MRM for the AGT marker peptide from the plasma digest, with peptide standard spiked into plasma digest at the expected concentration of endogenous AGT (45 μg/mL [22]). Recovery was calculated using the equation below:

$$ \mathrm{recovery}=\frac{\mathrm{plasma}\ \mathrm{AGT}}{\mathrm{plasma}\ \mathrm{spiked}\ \mathrm{with}\ \mathrm{AGT}\ \mathrm{peptide}\ \mathrm{standard}-\mathrm{plasma}\ \mathrm{AGT}}\times 100\% $$

The linearity of the method was evaluated using an eight-point calibration line (5 to 400 nM) in plasma chymotryptic digest using the internal standard method.

To confirm that the method delivered an effective ratio of the reduced and oxidised forms of AGT, human plasma was spiked with two precise ratios of the oxidised:reduced AGT (50:50 and 60:40; similar to the reported ratios in plasma) using human recombinant glycosylated AGT (3.4 mg/mL in TRIS buffer (pH 7.4)) expressed in HEK293 cells (all cysteines are in the oxidised form) which was kindly supplied by MRC group from Cambridge University (Prof. R. Carrell and Dr. R. Read, Cambridge Institute for Medical Research). To obtain the reduced form of the protein, human recombinant AGT standard was fully reduced with 50 mM DTT then alkylated with 200 mM IAM. Both reactions were carried out at 37 °C for 30 min in the dark using 10 kDa Amicon Ultra 0.5. The reduced protein sample was washed twice with 400 μL of TRIS buffer (pH 7.4) at 14,000×*g* for 7 min to remove excess reagents.

Figure 2 summarises the workflow followed in this study.

## Results and discussion

### Analytical strategy

To achieve successful MS analysis and accurate protein redox measurements, signature peptides should be reproducibly detected [23], and the alkylating agent used to modify the free thiol of the Cys peptides should have high reactivity, to ensure reaction completion, without negatively affecting the ionisation and fragmentation efficiency of the peptide [24]. Therefore, our method development strategy began by optimising a digestion protocol for the detection of the key Cys18 and Cys138 peptides, using samples of pure recombinant human AGT. Different strategies for protein reduction, alkylation and digestion were evaluated to achieve complete AGT digestion and maximum protein recovery with reliable detection of the modified Cys peptides. The performance of the deglycosylation enzyme PNGase F was assessed using SDS-PAGE.

Initial investigations revealed the reproducible detection of the two key modified Cys peptides with in-solution digestion using chymotrypsin as proteolytic enzyme and IAM as alkylating agent (see Electronic Supplementary Material (ESM) Fig. S1). The identities of the IAM-modified Cys peptides were confirmed by both high-resolution accurate mass (LC-MS) and peptide fragmentation pattern (LC-MS/MS) (see ESM Figs. S1 and S2) while complete deglycosylation was confirmed by band shift on SDS-PAGE (see ESM Fig. S3).

Further work reported below is based on the development of these initial findings.

#### Identification of the AGT marker peptide

During our initial experiments with recombinant AGT, a seven amino acid peptide, SVTQVPF, unique in sequence to AGT was identified from the AGT chymotryptic digest (see ESM Fig. S2). It was detected as a singly charged ion, having high signal intensity (higher than both Cys peptides) and mass accuracy < 1 ppm, making it ideally suited as a signature peptide for the absolute quantification of the total AGT in the plasma. Furthermore, SVTQVPF shows good electrospray ionisation and does not contain amino acids which are susceptible to modification, and we refer to SVTQVPF as the ‘AGT marker peptide’ henceforth.

### Detection of the oxidised and reduced forms of AGT in human plasma

With human plasma, a method for the selective enrichment of AGT was developed followed by differential alkylation and digestion of the sample using the optimised protocol. Targeted LC-MS/MS method working on the MRM mode was used to detect the key Cys peptides as well as the marker peptide from the plasma digest (Fig. 2). Combining differential alkylation of the sample with targeted MS/MS analysis was first used by Held et al. [12] to quantify the percent oxidation of targeted Cys from a cellular extract. They termed this approach OxMRM; quantitative cysteine oxidation analysis by MRM.

#### Selective enrichment of AGT from human plasma using ConA/RP-SPE

**Fig. 3:**
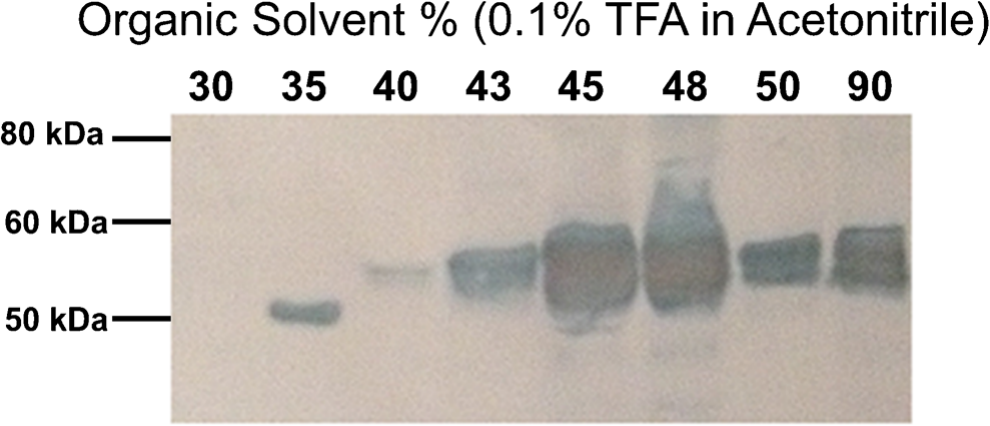
Western blotting of human AGT in the eight RP-SPE fractions using mouse anti-human serpin A8/angiotensinogen antibody. Plasma glycoproteins were enriched with ConA then fractionated using RP-SPE where proteins were eluted sequentially with step incrementing concentrations of acetonitrile in 0.1% TFA in water. High recovery of plasma AGT was identified, with the majority eluted at 43 to 50% acetonitrile

In identifying AGT eluting fractions, Western blotting revealed AGT detection in more than one fraction, with the majority eluting at 45% and 48% (*v*/*v*) acetonitrile in 0.1% TFA water (Fig. 3). Elution of the protein at a range of organic solvent (acetonitrile in 0.1% TFA) composition is to be expected with glycoproteins due to the glycan heterogeneity [25, 26]. Human AGT is known to have heterogeneous glycosylation [27] and so, the result of Western blotting is considered consistent with glycoprotein heterogeneity and previous studies conducted on AGT glycosylation.

Reversed-phase fractionation of the lectin affinity extracted glycoproteins using eight incrementing concentrations of acetonitrile was only necessary for method optimisation purposes. Following the results of Western blotting (Fig. 3), in actual plasma samples, AGT was subsequently extracted applying just two-step increments of acetonitrile mobile phase (acetonitrile in 0.1% TFA water (*v*/*v)*). In the first step, 40% acetonitrile, where very minimal AGT was evident, was used to wash the undesired proteins and the other matrix components to waste. In the second step, 50% acetonitrile was used to collect the remaining proteins (in fractions 43, 45, 48 and 50%), which yielded the majority of the AGT.

Reportedly, AGT has been detected directly from the plasma digest without applying any enrichment or depletion approaches [23, 28–31]; however, different depletion strategies can significantly improve the detection limit and the reproducibility of the methods [29, 31]. AGT has been identified with other plasma glycoproteins by using a multi-lectin approach [30, 32], and with commercial immune depletion columns, MARS-7 and MARS-14 [31], ProteoPrep20 [33] and Seppro® IgY12 coupled with multi-lectins [34]. Although the previously mentioned enrichment strategies, especially immunoaffinity depletion columns, offer high reproducibility and improved in-depth analysis for moderately and low abundant proteins, they are generally associated with high costs and require long processing times, and there are limitations associated with non-specific protein binding [31]. Our method achieved a quick and effective extraction of AGT, as only a two-step RP-SPE purification was required to capture the majority of the ConA retained AGT. Therefore, high recovery and specificity were achieved without reducing throughput, and a larger number of samples could be processed at a lower cost per sample compared with immunoaffinity depletion.

#### Detection of the oxidised and reduced forms of AGT using differential alkylation and targeted LC-MS/MS

**Fig. 4:**
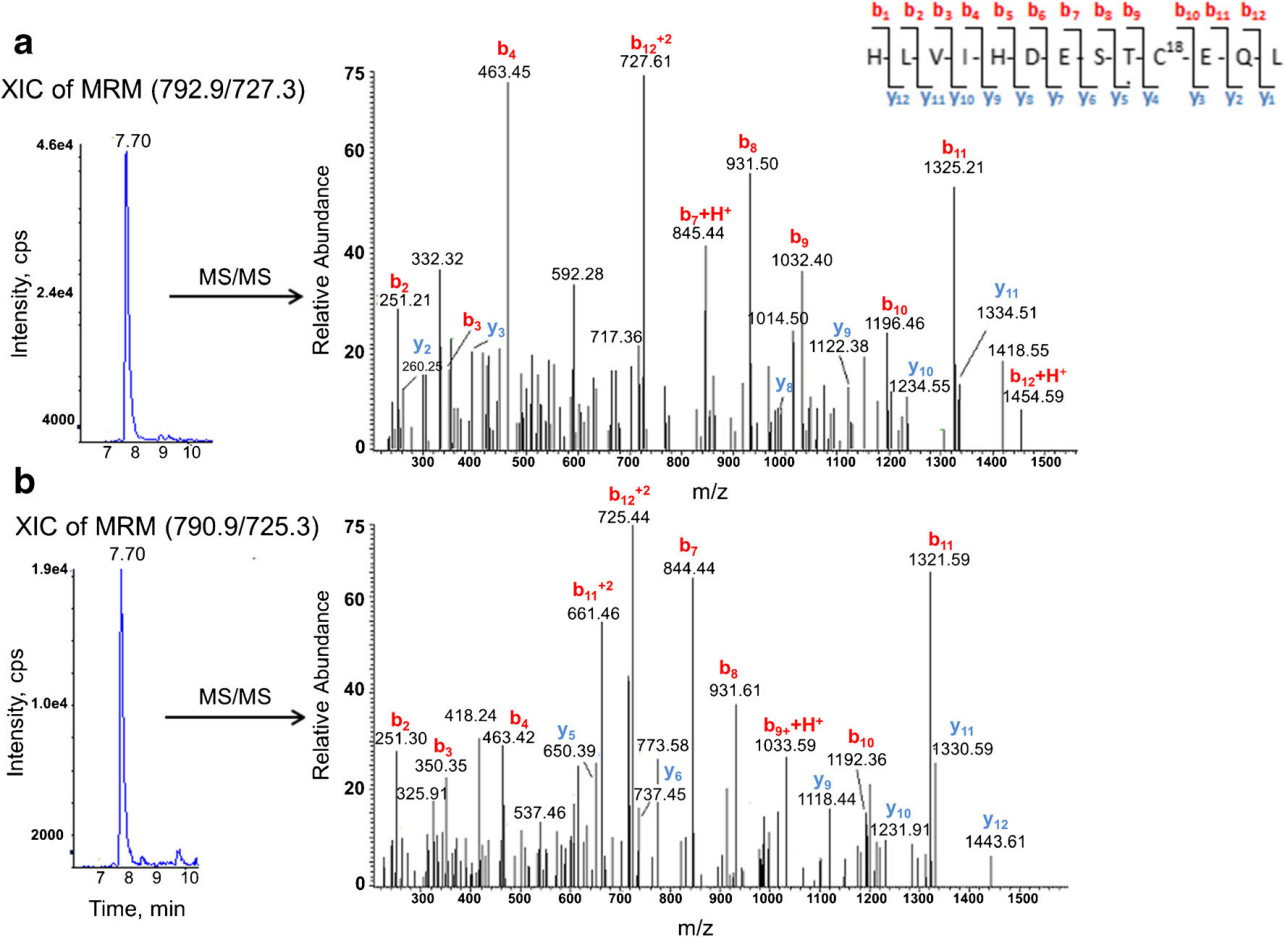
The extracted ion chromatogram (XIC) of MRM and the corresponding MS/MS spectra for the differentially alkylated Cys18 peptides detected in a typical human plasma digest. **a** Cys18 peptide alkylated with isotope-labelled ^13^C_2_,D_2_-iodoacetamide representing the oxidised form of AGT in the plasma. **b** Cys18 peptide alkylated with unlabelled ^13^C_0_,D_0_-iodoacetamide representing the reduced form of AGT in the plasma. Peptide identity and differential alkylation were confirmed by MS/MS spectra

**Fig. 5:**
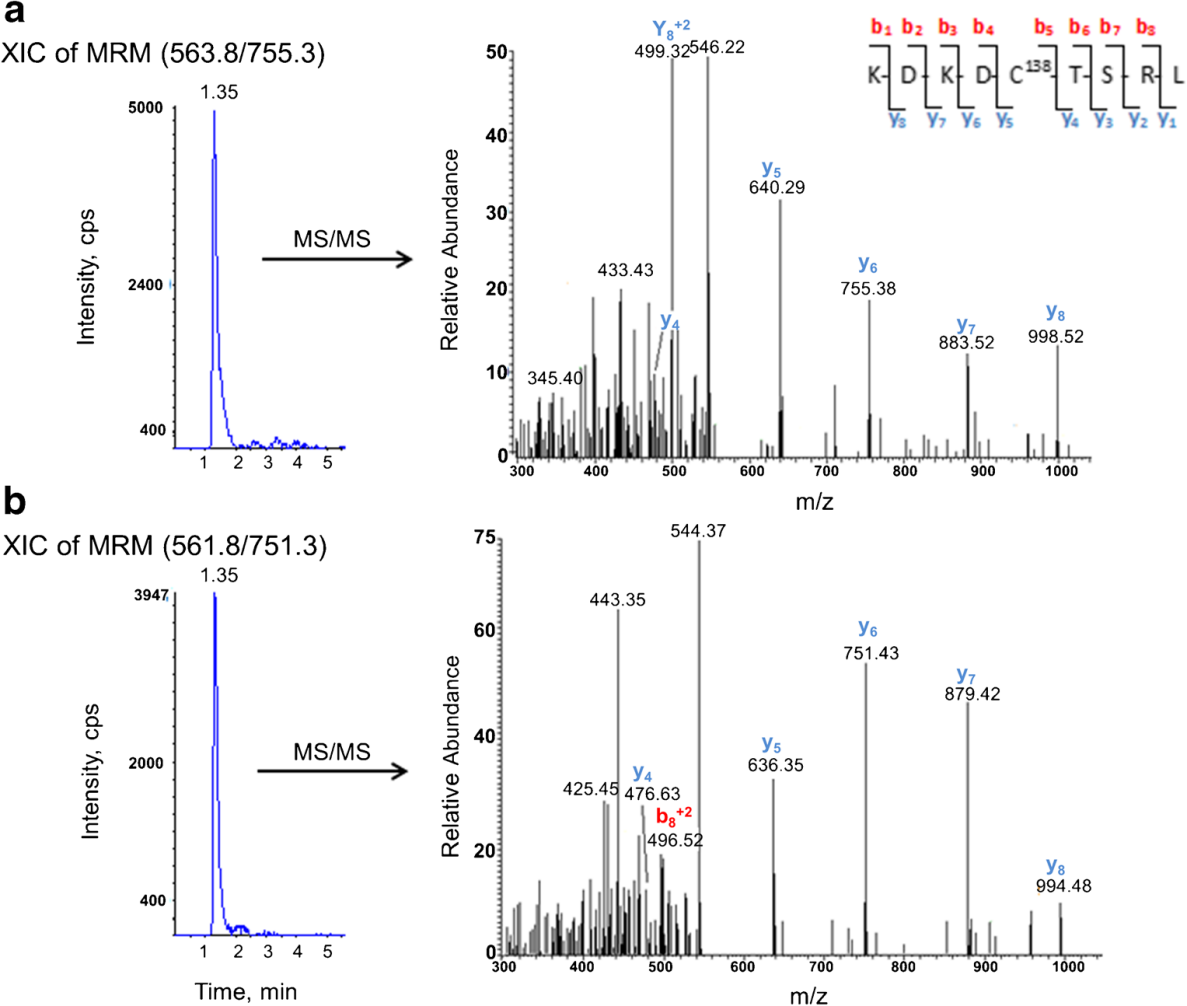
The XIC of MRM and the corresponding MS/MS spectra for the differentially alkylated Cys138 peptides detected in the plasma digest. **a** Cys138 peptide alkylated with isotope-labelled ^13^C_2_,D_2_-iodoacetamide representing the oxidised form of AGT in the plasma. **b** Cys138 peptide alkylated with unlabelled ^13^C_0_,D_0_-iodoacetamide representing the reduced form of AGT in the plasma. Peptide identity and differential alkylation were confirmed by MS/MS spectra

**Fig. 6:**
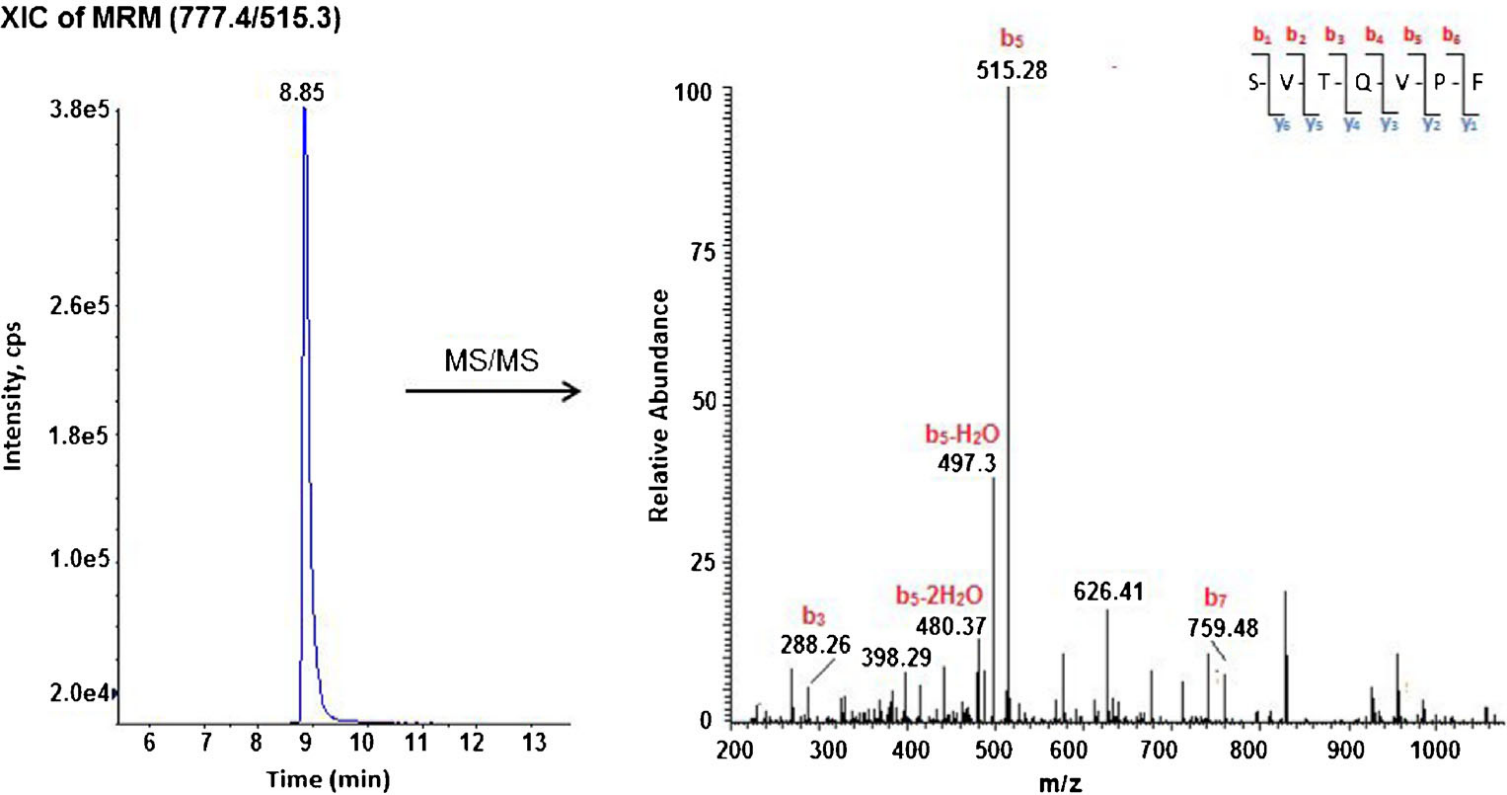
The XIC of MRM and the corresponding MS/MS spectrum for AGT marker peptide detected in the plasma digest of a typical human plasma sample. Peptide identity was confirmed by MS/MS spectrum

The two distinct redox forms of AGT were detected in human plasma using the developed targeted LC-MS/MS workflow and applying the differential alkylation approach. Differentially alkylated Cys18 and Cys138 peptides and AGT marker peptide with their corresponding MS/MS spectra are presented in Figs. 4, 5 and 6 respectively.

The modified Cys18 peptide showed a higher MS response and better *S*/*N* ratio compared to the modified Cys138 peptide (Figs. 4 and 5); however, the highest signal intensity was observed with AGT marker peptide MRM (Fig. 6). This is consistent with our previous findings using the pure recombinant AGT and with the results obtained from the three peptide standards (see ESM Fig. S4). The different MS response among the three peptides is not unexpected. The relative ionisation and fragmentation of the peptide are dependent on the sequence, other co-eluting ions and the presence of post translation modification [25].

With regard to the differentially alkylated Cys peptides, alkylation with ^13^C_2_,D_2_-IAM introduced a 4-Da increase in the mass of the singly charged Cys peptides (2 *m*/*z* for doubly charged peptides) compared to the unlabelled alkylated peptide (Figs. 4 and 5). This made the two plasma AGT forms mass distinguishable when analysed using the targeted LC-MS/MS method. The differentially alkylated Cys peptides, which are signatures of the oxidised and the reduced forms of plasma AGT, exhibited similar physicochemical properties and therefore were eluted at the same retention time (Figs. 4 and 5). This ensured that the two peptide forms experienced a similar chemical environment upon analysis, which is crucial for accurate quantification.

Of importance, both Cys peptides have a potential asparagine (N) glycosylation site in their sequences: HLVIH**N**ESTC^18^EQL and KDK**N**C^138^TSRL, and so, effective deglycosylation was essential for two reasons: firstly, to achieve efficient digestion and release of the key peptides since glycosylation can inhibit protease processing. Secondly and more importantly, it is required to attain the correct parent mass of the peptide in Q1 and thus have the correct mass for MRM transitions. Upon oligosaccharide cleavage using PNGase F, asparagine is converted to aspartic acid (D) resulting in approximately 1 and 0.5 *m*/*z* increase in the singly and doubly charged peptide ions respectively [25]. Since both modified Cys peptides are doubly charged, then a further 0.5 *m*/*z* was added to the original *m*/*z* to achieve accurate detection of peptides from plasma.

#### Confirmation of identities of peptides derived from plasma AGT by LC-MS/MS

Even though MRM signals are highly analyte specific, and the MRM transitions for each peptide were detected at the same retention times as the corresponding standard, the identities of the differentially alkylated Cys peptides and AGT marker peptide derived from plasma were further confirmed using LC-MS/MS (Figs. 4, 5 and 6). The amino acid sequence of detected peptides was interpreted using comprehensive *y*- and *b*-ion series for Cys18 (Fig. 4) and mainly *y*-ion for Cys138 (Fig. 5), additionally both spectra were similar to their corresponding standards (see ESM Fig. S5). Poor fragmentation was noticed with the marker peptide (Fig. 6), but was still comparable with the standard (see ESM Fig. S5). The selected product ions in MRM transitions for each peptide were apparent in the MS/MS spectra.

### Method performance

A reliable level of 43% (± 2.0%, *n* = 5) was achieved for the overall recovery of AGT from the plasma. Calculation of the variability of the method revealed an acceptable analytical precision (CV% below 15%) for the two differentially alkylated Cys18 peptides and the AGT marker peptide (Table 2), which make them suitable for quantitative protein measurement in clinical samples in line with the FDA guidelines for bioanalytical method validation [21]. Differentially alkylated Cys138 peptides were associated with low signal intensity and high variability and were not reproducibly detected between replicates (Table 2) and, therefore, were deemed not suitable for the quantification of the redox forms of AGT in the plasma. Hence, the differentially alkylated Cys18 peptides can be used to infer the oxidation level of AGT while the marker peptide can be used for the measurement of the total AGT in the plasma. To confirm that the method delivered an effective ratio of the reduced and oxidised forms of AGT, we spiked human plasma with two precise ratios of the oxidised:reduced AGT (50:50 and 60:40; similar to the reported ratios in plasma) and were able to reproduce these ratios when the samples were analysed using the developed method (50.3% ± 1.9% and 58.9% ± 1.6% representing plasma sample spiked with 50 and 60% oxidised AGT respectively, *n* = 3). The MRM assays were free of potential interfering substances at the retention times of the peptides, and no additional peaks were observed when analysing blank plasma samples, which make the MRM assay specific and selective for Cys18 and marker peptides.

**Table 2:** The analytical precision of the developed targeted analysis for plasma-derived AGT

Protein	AGT signature peptide	Peptide transition	CV% (*n* = 6)
Oxidised AGT	Cys18 peptide alkylated with ^13^C_2_,D_2_-IAM	792.9/727.3	11.1
	Cys138 peptide alkylated with ^13^C_2_,D_2_-IAM	Sum of three transitions^a^	18.7
Reduced AGT	Cys18 peptide alkylated with ^13^C_0_,D_0_-IAM	790.9/725.3	9.6
	Cys138 peptide alkylated with ^13^C_0_,D_0_-IAM	Sum of three transitions^b^	27.2
Total AGT	Marker peptide	777.4/515.3	6.8

^a^The transitions are 563.8/640.6, 563.8/755.4 and 563.8/883.4

^b^The transitions are 561.8/636.5, 561.8/751.3 and 561.8/879.5

**Fig. 7:**
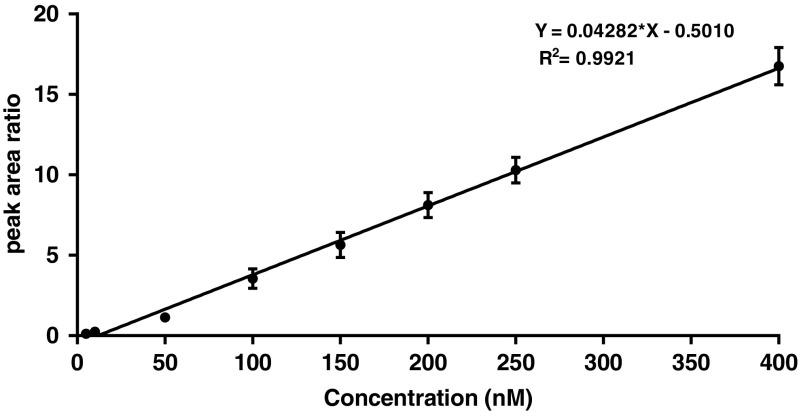
The linear response of the total AGT assay. Known quantities of AGT marker peptide standards were spiked in the plasma chymotryptic digest to generate a calibration plot from 5 to 400 nM. The ratio between the peak areas of the marker peptide standard and the internal standard (2 μM NEM alkylated Cys18 peptide) was plotted against AGT marker peptide standard concentrations. The regression line of the marker AGT peptide showed a linear response which extends to the low nanomolar concentrations with a correlation coefficient of *R*^2^ = 0.992. Error bars represent the SD of three technical replicates

With regard to the assay of total AGT in the plasma, the limit of detection (LOD) and the lower limit of quantification (LLOQ) of the method were 0.5 and 5 nM respectively. The method showed a linear response in the nanomolar range with a high correlation coefficient (*R*^2^ = 0.992) as seen in the calibration curve in Fig. 7. Analytical throughput requires overnight protein digestion with 24 sample batches possible with an LC-MS/MS analysis time of 20 min/sample.

Our method demonstrates reproducible and quantitative analysis of these important AGT peptides and represents the first study to use MS workflow to detect the oxidised and the reduced forms of AGT in human plasma. The two previous studies that quantified the redox switch of AGT in the plasma of pre-eclamptic women used either Western blotting or ELISA [3, 8]. The novel MS workflow we followed is considered a more reliable quantitative methodology than Western blotting, used by Zhou et al. [3], which is at best semi-quantitative. Our developed MS-based methodology can be applied to accurately quantify the oxidation level of AGT in clinical samples as a percentage of the sum of the reduced and oxidised AGT forms. By using this methodology, we also confirm that changes in AGT levels correlate to changes in oxidation state rather than total level. This provides more accurate measurements than the ELISA developed by Rahgozar et al. [8] where they only detected the reduced form of AGT, and reported its level as a percentage of that observed in a pooled standard rather than that of the sum of the two forms of the protein.

## Conclusions

The current work represents the first study to use an MS workflow to measure the oxidised and reduced forms of AGT in human plasma. Our methodology uses high-throughput two-dimensional chromatographic purification and a differential alkylation strategy coupled with targeted LC-MS/MS to provide a more reliable and selective quantitative approach than the previously reported antibody-based assays used to measure the oxidation level of AGT.

Additionally, our method offers several advantages over previous redox proteomic studies. It avoids the use of sizable costly alkylating reagents (e.g. ICAT, biotinylated IAM) which can limit effective alkylation and the use of highly denaturing conditions that can facilitate the breakdown of the disulphide linkage leading to inaccurate quantification. Additionally, the use of conventional LC-MS/MS as opposed to a nano-LC system typically used to analyse proteins in biological samples, makes the method both more applicable to routine use when high-throughput analysis is required (20 min run time instead of hours with nano-LC systems), and more transferable to laboratories not equipped with a nano-LC system. The extensive sample preparation resulted in only 43% recovery. However, the workflow was reproducible and allowed reliable detection of the signature AGT peptides.

It should be noted that the high complexity of the plasma (~ 3.3 mg proteins/50 μL plasma) and the presence of sugar moiety attached to the asparagine residue in both Cys peptides retarded early effective alkylation directly from the plasma; consequently, differential alkylation was achieved after protein enrichment and deglycosylation. Since differential alkylation was performed following sample preparation, then the measured levels of oxidation may not accurately reflect those of AGT in the plasma. However, the developed method will be applied to compare the relative oxidation level of the protein between samples from healthy women and those with developing pre-eclampsia rather than measuring the actual oxidation level of AGT. In this case, the reproducibility of the method is paramount, and the demonstrated methodology showed acceptable analytical precision. This provides a good level of confidence to move towards the clinical application of the methodology to quantify the oxidation and total AGT levels in samples collected from pre-eclamptic and normotensive women.

## Electronic supplementary material

ESM 1 (PDF 355 kb)
