# Supplementary material for: Measurement of the total angiotensinogen and its reduced and oxidised forms in human plasma using targeted LC-MS/MS
Source: Anal Bioanal Chem. 2018 Nov 21;411(2):427–37. doi: 10.1007/s00216-018-1455-2 (PMC6336742; doi:10.1007/s00216-018-1455-2)
Supplement: Supplementary file 1 — (PDF 355 kb) [file 216_2018_1455_MOESM1_ESM.pdf]

Analytical and Bioanalytical Chemistry

Electronic Supplementary Material

Measurement of the total angiotensinogen and its reduced and oxidised forms in human plasma using targeted LC-MS/MS

Lina A. Dahabiyeh, David Tooth, Robin W. Carrell, Randy J. Read, Yahui Yan, Fiona Broughton Pipkin, David A. Barrett

a) Chymotrypsin digestion

MRKRAPQSEM	APAGVSLRAT	ILCLLAWAGL	AAGD**RVYIHP**	F**HLVIHNEST**
**C^{18}EQL**AKANAG	KPKDPTFIPA	PIQAKTSPVD	EKALQDQL**VL**	**VAAKLDTEDK**
**L**RAAMVGMLA	NFLGFRIY**GM**	**HSELWGVVHG**	**ATVL**SPTAVF	GTLASLYL**GA**
**LDHTADRLQA**	**ILGVPWKDKN**	**C^{138}TSRLDAHKV**	**LSALQAVQGL**	**LVAQGRADSQ**
**AQLLLSTVVG**	**VFTAPGLHL**K	QPFVQGLALY	T**PVVLPRSLD**	**FTELDVAAEK**
**IDRFMQAVTG**	**WKTGCSL**MGA	SVDSTLAF**NT**	**YVHF**QGKMKG	FSL**LAEPQEF**
WVDNSTSVSV	PMLSGMGTF**Q**	**HWSDIQDNF**S	**VTQVPFTESA**	**CLLLIQPHY**A
SDLDKVEGLT	FQQNSLNWMK	KL**SPRTIHL**T	MPQLVLQGSY	DLQDLL**AQAE**
**LPAIL**HTELN	LQKL**SNDRIR**	**VGEVLNSIFF**	**ELEADEREPT**	**ESTQQLNKPE**
**VLEVTLNRPF**	LF**AVYDQSAT**	**ALHFLGRVAN**	**PLSTA**	

b) Trypsin digestion

MRKRAPQSEM	APAGVSLRAT	ILCLLAWAGL	AAGD**RVYIHP**	FHLVIHNEST
C^{18}EQLAK**ANAG**	**KPKDPTFIPA**	**PIQAKTSPVD**	**EKALQDQLVL**	**VAAKLDTEDK**
**LR**AAMVGMLA	NFLGFRIYGM	HSELWGVVHG	ATVLSPTAVF	GTLASLYLGA
LDHTADR**LQA**	**ILGVPWKDKN**	**C^{138}TSRLDAHKV**	**LSALQAVQGL**	**LVAQGR**ADSQ
AQLLLSTVVG	VFTAPGLHLK	**QPFVQGLALY**	**TPVVLPRSLD**	**FTELDVAAEK**
IDR**FMQAVTG**	**WK**TGCSLMGA	SVDSTLAFNT	YVHFQGKMKG	FSLLAEPQEF
WVDNSTSVSV	PMLSGMGTFQ	HWSDIQDNFS	VTQVPFTESA	CLLLIQPHYA
SDLDKVEGLT	FQQNSLNWMK	KLSPRTIHLT	MPQLVLQGSY	DLQDLLAQAE
LPAILHTELN	LQKLSNDRIR	**VGEVLNSIFF**	**ELEADER**EPT	ESTQQLNKPE
VLEVTLNRPF	LFAVYDQSAT	ALHFLGR**VAN**	**PLSTA**	

Fig. S1 AGT peptide sequence coverage from LC-MS/MS data analysed by Mascot for chymotrypsin digest (a) and trypsin digest (b). Matched peptides are shown in red. Detected Cys18 and Cys138 peptides are underlined. Sequence starts from RVYI…. presented in bold black letters, the first 33 amino acids are for the signal peptide

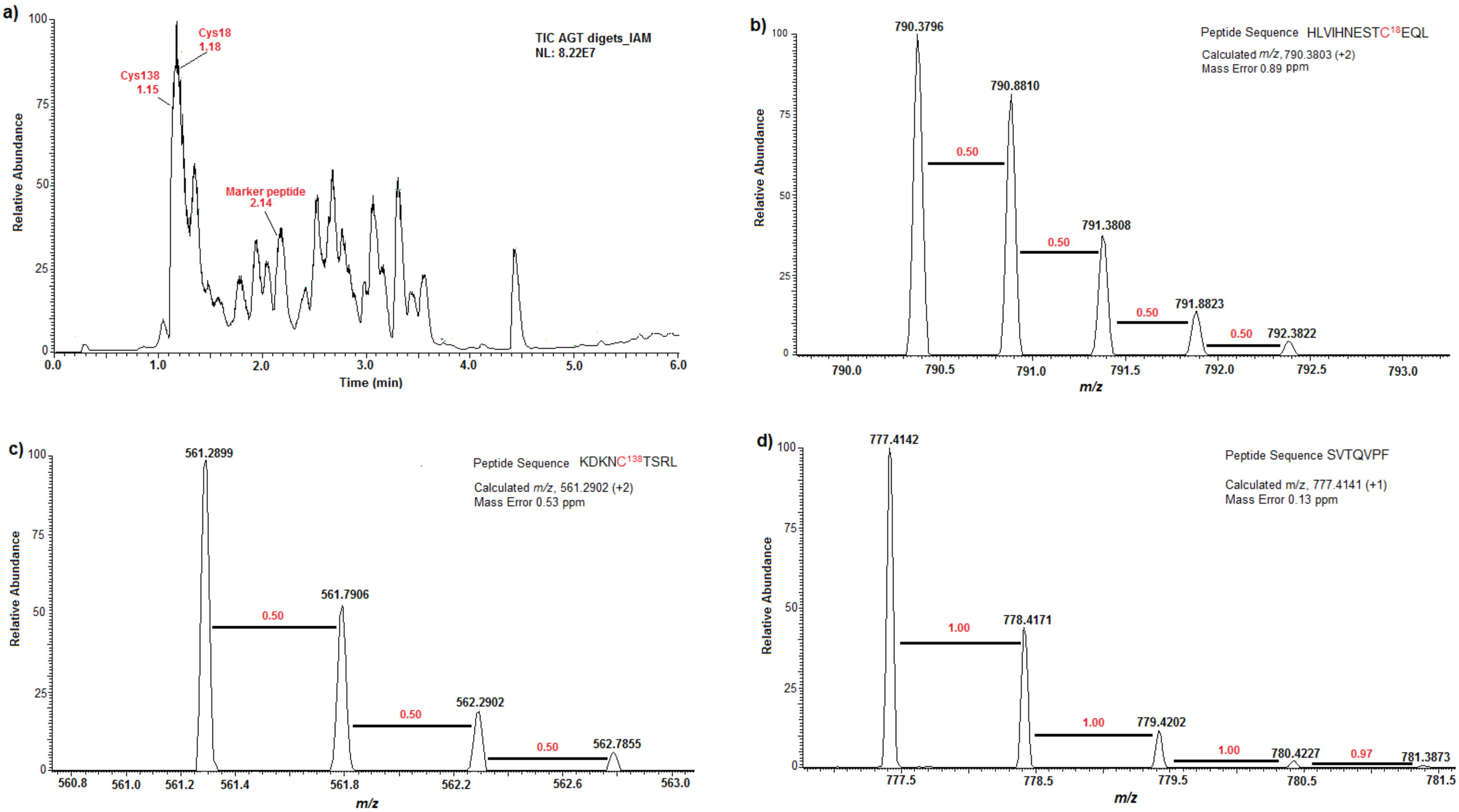

Fig. S2 Analysis of peptides derived from chymotryptic digest of recombinant human AGT by high resolution accurate mass. (a) Total ion chromatogram (TIC), (b) $[M+2H]^{2+}$ Ion signal of iodoacetamide alkylated Cys18 peptide, (c) $[M+2H]^{2+}$ Ion signal of iodoacetamide alkylated Cys138 and (d) $[M+1H]^{+}$ ion signal of AGT marker peptide, all extracted from the TIC

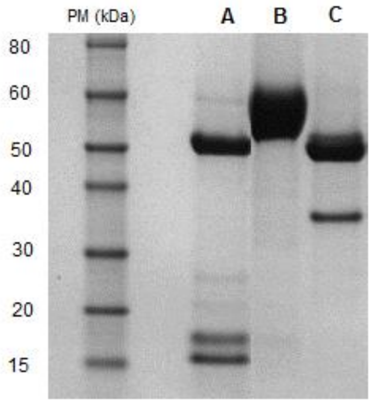

Fig. S3 Coomassie blue stained 1D SDS-PAGE for human recombinant AGT treated with PNGase F. A, B and C represent unglycosylated AGT, glycosylated AGT, and glycosylated AGT after deglycosylation with PNGAse F. After deglycosylation, glycosylated AGT band at 60 kDa disappeared and a new band appears at the same mass of the unglycosylated AGT (around 53 kDa) indicating full deglycosylation was achieved. The band at 36 kDa refers to PNGase F

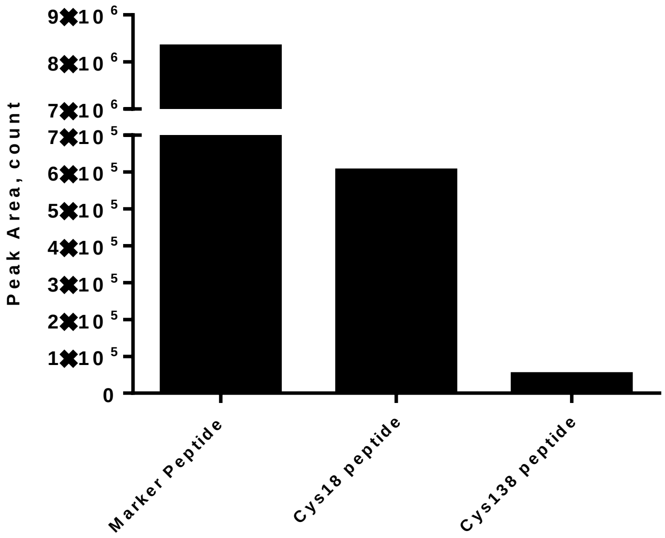

Fig. S4 Different MS responses of 0.5 µM standards of marker peptide, iodoacetamide alkylated Cys18 and iodoacetamide alkylated Cys138 peptides analysed by targeted LC-MS/MS. AGT marker peptide showed the highest MS response while iodoacetamide alkylated Cys138 showed the lowest MS response. The marker peptide showed ~13x higher peak area than Cys18 which in turn displayed ~12x higher response than Cys138 peptide

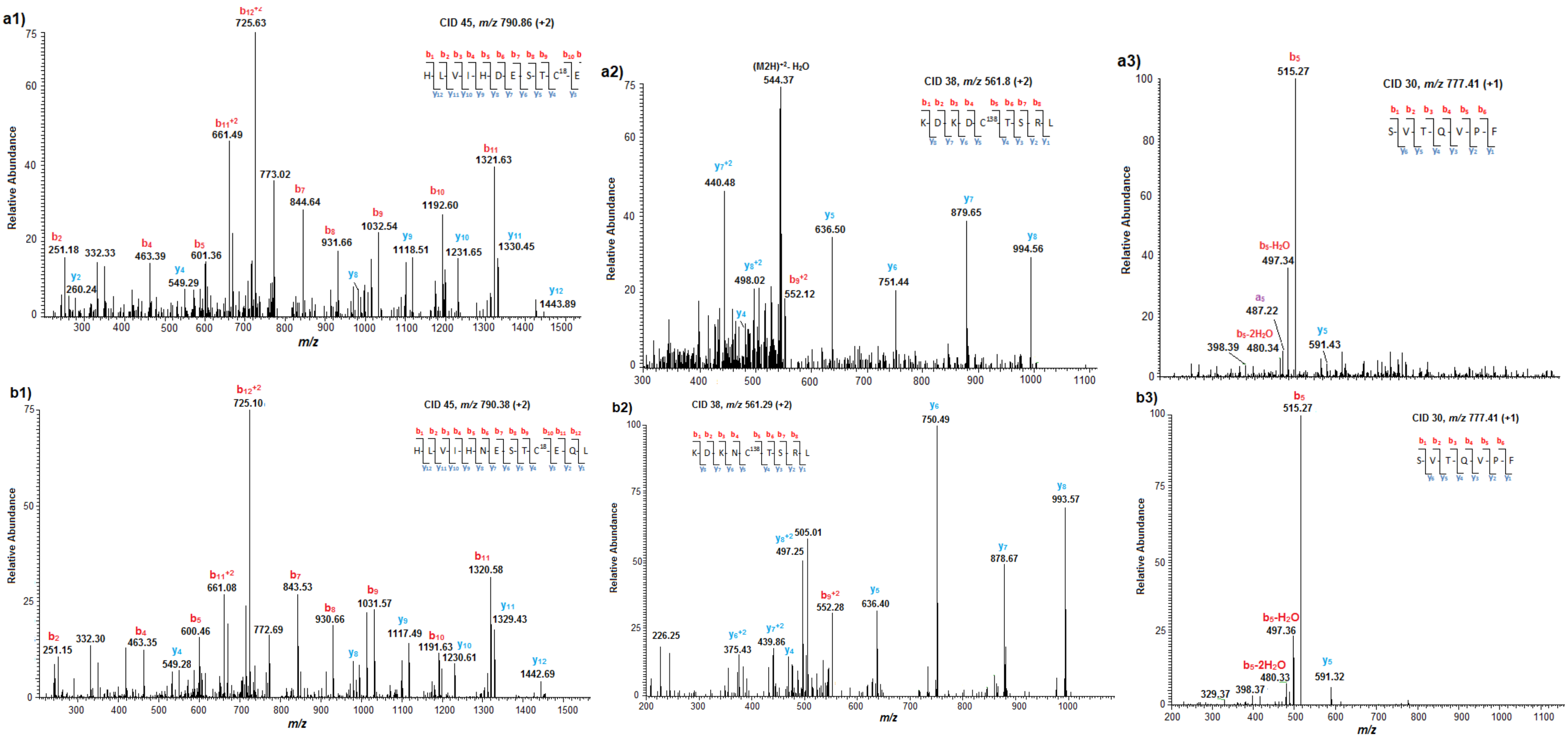

Fig. S5 (Above) the averaged MS/MS spectra for the IAM modified Cys18, Cys138 and marker peptides detected from plasma chymotryptic digest (a1, a2 and a3 respectively). (Below) the averaged scan MS/MS spectra for the corresponding IAM modified Cys18, Cys138 and marker peptide standards (b1, b2 and b3). The acquired spectra were comparable to their corresponding standards confirming the identity of the signature AGT peptides derived from human plasma
